# Cellular transcripts regulated during infections with Highly Pathogenic H5N1 Avian Influenza virus in 3 host systems

**DOI:** 10.1186/1743-422X-8-196

**Published:** 2011-04-29

**Authors:** Vinod RMT Balasubramaniam, Sharifah S Hassan, Abdul R Omar, Maizan Mohamed, Suriani M Noor, Ramlan Mohamed, Iekhsan Othman

**Affiliations:** 1 Infectious Disease Laboratory (MR3), School of Medicine and Health Sciences, Monash University, Sunway Campus, Kuala Lumpur, Malaysia; 2 Institute of Bioscience, University Putra Malaysia, UPM Serdang, Selangor, 43400, Malaysia; 3 Veterinary Research Institute, 31400 Ipoh, Perak, Malaysia

**Keywords:** Avian Influenza virus, mRNA differential display, annealing control primer, hsp60 gene, cyclin D2 gene, Interleukin 8 gene

## Abstract

**Background:**

Highly pathogenic Avian Influenza (HPAI) virus is able to infect many hosts and the virus replicates in high levels in the respiratory tract inducing severe lung lesions. The pathogenesis of the disease is actually the outcome of the infection as determined by complex host-virus interactions involving the functional kinetics of large numbers of participating genes. Understanding the genes and proteins involved in host cellular responses are therefore, critical for the elucidation of the mechanisms of infection.

**Methods:**

Differentially expressed transcripts regulated in a H5N1 infections of whole lung organ of chicken, *in-vitro *chick embryo lung primary cell culture (CeLu) and a continuous Madin Darby Canine Kidney cell line was undertaken. An improved mRNA differential display technique (Gene Fishing™) using annealing control primers that generates reproducible, authentic and long PCR products that are detectable on agarose gels was used for the identification of differentially expressed genes (DEGs). Seven of the genes have been selected for validation using a TaqMan^® ^based real time quantitative PCR assay.

**Results:**

Thirty seven known and unique differentially expressed genes from lungs of chickens, CeLu and MDCK cells were isolated. Among the genes isolated and identified include heat shock proteins, Cyclin D2, Prenyl (decaprenyl) diphosphate synthase, IL-8 and many other unknown genes. The quantitative real time RT-PCR assay data showed that the transcription kinetics of the selected genes were clearly altered during infection by the Highly Pathogenic Avian Influenza virus.

**Conclusion:**

The Gene Fishing™ technique has allowed for the first time, the isolation and identification of sequences of host cellular genes regulated during H5N1 virus infection. In this limited study, the differentially expressed genes in the three host systems were not identical, thus suggesting that their responses to the H5N1 infection may not share similar mechanisms and pathways.

## Background

Avian Influenza virus (AIV) is a member of the *Orthomyxoviridae *family of negative-stranded, segmented RNA viruses and represents a particularly attractive model system as viral replication strategies are closely intertwined with normal cellular processes including the host defense and stress pathways [1]. Over the course of evolution, Influenza virus has developed translational control strategies that utilize cap-dependent translation initiation mechanisms. This causes the host-cell proteins to preferentially synthesize viral proteins and prevent the activation of antiviral response. Translational regulation is a critical component of the cellular response to a variety of stimuli, including growth promoting and growth-repressing signals. Similarly, the cellular response to stress, such as viral infection, nutrient deprivation, accumulation of misfolded proteins and ER stress, and finally heat shock involves translational control mechanisms that function to activate and repress mRNA translation depending on environmental conditions. For example, during Influenza virus infection, there is a dramatic shutoff of cellular protein synthesis and the selective translation of viral mRNAs [1-3]. Concurrently, in heat shock or stressed cells, there is similarly a disruption of 'normal' cellular protein synthesis and a subsequent redirection of translation to heat shock mRNAs [4-6]. This clearly shows that the Influenza virus infections on cells are closely intertwined with normal cellular processes, including host defense and stress pathways.

The widespread distribution of highly pathogenic avian H5N1 Influenza A viruses in wild birds and, in particular, in domestic poultry populations continues to pose a threat to public health. Severe respiratory disease and a high case-fatality rate have become a hallmark of H5N1 infection in humans as well as in other mammalian species [7-10]. To develop efficient therapeutics against this virus, understanding how the virus interacts with the host in natural infection is necessary. Having insights into the host's responses to influenza (H5N1) would help define targets for therapeutic intervention [11].

One way to do this is to elucidate the mechanisms of virus pathogenesis in chickens, however, how host cells interact and the molecular mechanisms underlying the pathophysiologic process of HPAIV infection in chicken is still poorly understood. Still lacking, also, are the first hand information on the molecular changes in the host induced by the virus to promote its replication and also the pathways triggered in the host that result in immunity and or clearance of the viral infection [11]. The outcome of the infection is determined by complex host-virus interactions with a large number of altered transcriptional and translational rates, and functional kinetics of participating genes. An overview of host responses to AIV at transcriptional level in the trachea and lungs induced by H9N2, H3N2 and H1N1 infection have described the involvement of many genes involved in innate immunity, interleukin activity and vesicle trafficking such as endocytosis and phagocytosis during virus entry [12-15].

We undertook the present study as a preliminary work to understand the selective transcriptome which were up regulated and down regulated during the time of infection with 3 different types of hosts i.e. MDCK cells, primary CeLu cells and lung tissues of infected chickens. We employed a new differential display GeneFishing™ PCR technique to compare the gene expression in normal and infected cells and tissues. This sensitive technique is based on the determination of multiple expression patterns of pre-determined sequences and we also combined it with the use of annealing control primer (ACP)™ technology in order to provide a primer with annealing specificity to the template, and allow only targeted product to be amplified without any false artifacts [16,17]. The other great advantage of this technique is that the bands can be isolated and the genes cloned in a vector for sequence identification and stored for further use.

## Materials and methods

### Viruses

Avian Influenza virus, isolate A/chicken/Malaysia/5744/2004 H5N1 was provided by Veterinary Research Institute, Ipoh, Perak, Malaysia. This virus was confirmed to be highly pathogenic in chickens via the intra cerebral pathogenicity test (conducted at the OIE, World Avian Influenza Reference Centre of the Australian Animal Health Laboratory, Geelong, Australia). The pathogenicity of the virus was also confirmed by the demonstration of multiple basic amino-acid sequence at the cleavage site of the HA gene. The viruses were initially isolated and passaged in Madin-Darby canine kidney (MDCK) cells. The virus stock was aliquoted, and titrated to determine tissue culture infection dose 50% (TCID_50_) in MDCK cells. The experiments were carried out in a Bio-safety level 3 (BSL-3) facility at the Veterinary Research Institute, Ipoh, Perak, Malaysia.

### Chickens and virus infection

Six specific pathogen free (SPF) chickens of one-week of age were each infected intranasally with allantoic fluid containing 10^4 ^EID50/100 μl H5N1 virus. The chickens were kept in an isolator within a BSL-3 facility of the Veterinary Research Institute, Ipoh Malaysia with food and water available *ad libitum*. The same batch and age-matched control chickens were treated with PBS pH 7.2 used for virus dilution. As this virus is highly pathogenic to chickens, previous studies have shown that the virus was able to kill the chickens within 24-48 hrs post infection, depending on the dose. In this study, however, chickens were euthanized at 32 hrs post infection and the lungs harvested. Both the control and infected chickens were sacrificed at the same time. The lung tissues were kept at -80°C until used for total mRNA extraction. All animal studies were performed according to protocols approved by Animal Ethics committee of the Veterinary Research Institute (VRI) Malaysia, Department of Veterinary Sciences Malaysia.

### Cells and virus infection

Primary chicken embryo lung cells (CeLu) were prepared from 19-20 day old-embryos of SPF eggs. Cells were seeded at a density of 10^6^cells/ml in Dulbecco's Modified Eagle Medium (DMEM; GIBCO), supplemented with 10% fetal bovine serum (FBS; GIBCO), 100 units/ml penicillin (GIBCO), and 100 mg/ml streptomycin (GIBCO). MDCK (Madin Darby Canine Kidney) cells were also grown as monolayers in Minimum Essential Media (MEM; GIBCO), supplemented with 10% fetal bovine serum (FBS; GIBCO), and the same concentration of antibiotics as above. Confluent monolayer cells were infected with the isolate A/chicken/Malaysia/5744/2004 H5N1 at a multiplicity of infection of 5. Infected cells showing 5-10% cytopathic effect (within 32 hours) were harvested control and infected monolayer cells were washed twice with PBS pH 7.2, scraped into a 15 ml conical tube and centrifuged at 1,500rpm for 15 mins. The cell pellets were stored at -80°C or used immediately for total mRNA extractions.

### Messenger RNA isolation

mRNA was extracted from the infected and uninfected or control MDCK and primary CeLu cells, lungs of infected and control chickens using the RNeasy^® ^mini kit (QIAGEN Inc., Valencia, CA), according to the manufacturer's instructions.

### cDNA synthesis

The mRNA extracted was employed for the synthesis of first strand cDNA by reverse transcriptase, as described by Hwang et al., 2003. Reverse transcription was performed for 1.5 hour at 42°C in a final reaction volume of 20 μl containing 3 μg of the purified mRNA, 4 μl of 5× reaction buffer (Promega, Madison, WI), 5 μl of deoxyribonucleotide triphosphate (each 2 mmol), 2 μl of 10 μmol cDNA synthesis primer deoxythiamine annealing control primer 1 (dT-ACP1; Table 1), 0.5 μl of RNasin^® ^RNase Inhibitor (40 U/μl; Promega), and1 μl of Moloney murine leukemia virus reverse transcriptase (200 U/μl; Promega). First-strand cDNA was diluted by the addition of 80 μl of ultra-purified water for the GeneFishing™ PCR, and stored at -20°C until use.

**Table 1:** Primer sequence used in cDNA synthesis and ACP™-based PCR

Use	Primer name	Sequence
cDNA synthesis primer	dT-ACP1	5'-CTGTGAATGCTGCGACTACGATIIIII (T) 18-3'
Reverse primer	dT-ACP2	5'-CTGTGAATGCTGCGACTACGATIIIII (T) 15-3'
Arbitrary primer	ACP1	5'-GTCTACCAGGCATTCGCTTCATIIIIIGCCATCGACC-3'
(Forward primer)	ACP2	5'-GTCTACCAGGCATTCGCTTCATIIIIIAGGCGATGCC-3'
	ACP3	5'-GTCTACCAGGCATTCGCTTCATIIIIICCGGAGGATG-3'
	ACP4	5'-GTCTACCAGGCATTCGCTTCATIIIIIGCTGCTCGCG-3'
	ACP5	5'-GTCTACCAGGCATTCGCTTCATIIIIIAGTGCGCTCG-3'
	ACP6	5'-GTCTACCAGGCATTCGCTTCATIIIIIGGCCACATCG-3'
	ACP7	5'-GTCTACCAGGCATTCGCTTCATIIIIICTGCGGATCG-3'
	ACP8	5'-GTCTACCAGGCATTCGCTTCATIIIIIGGTCACGGAG-3'
	ACP9	5'-GTCTACCAGGCATTCGCTTCATIIIIIGATGCCGCTG-3'
	ACP10	5'-GTCTACCAGGCATTCGCTTCATIIIIITGGTCGTGCC-3'
	ACP11	5'-GTCTACCAGGCATTCGCTTCATIIIIICTGCAGGACC-3'
	ACP12	5'-GTCTACCAGGCATTCGCTTCATIIIIIACCGTGGACG-3'
	ACP13	5'-GTCTACCAGGCATTCGCTTCATIIIIIGCTTCACCGC-3'
	ACP14	5'-GTCTACCAGGCATTCGCTTCATIIIIIGCAAGTCGGC-3'
	ACP15	5'-GTCTACCAGGCATTCGCTTCATIIIIICCACCGTGTG-3'
	ACP16	5'-GTCTACCAGGCATTCGCTTCATIIIIIGTCGACGGTG-3'
	ACP17	5'-GTCTACCAGGCATTCGCTTCATIIIIICAAGCCCACG-3'
	ACP18	5'-GTCTACCAGGCATTCGCTTCATIIIIICGGAGCATCC-3'
	ACP19	5'-GTCTACCAGGCATTCGCTTCATIIIIITCTGCGAGC-3'
	ACP20	5'-GTCTACCAGGCATTCGCTTCATIIIIIGACGTTGGCG-3'

ACP, annealing control primer; the polydeoxyinosine [poly (dI)] linkers are underlined. In the table, "I" represents deoxyinosine

### Annealing control primer™-based GeneFishing™ PCR

Differentially expressed genes (DEGs) were screened by the annealing control primer (ACP) ™-based PCR method using the GeneFishing™ DEG kits (Seegene, Seoul, South Korea) [17]. The GeneFishing™ PCR technique involved an ACP™ system that had a unique tripartite structure in that its distinct 3'-end target core sequence and 5'-end nontarget universal sequence portions were separated by a regulator, it used primers that annealed specifically to the template, and it allowed only genuine products to be amplified; this process eliminates false positive results. Second-strand cDNA synthesis and subsequent PCR amplification were conducted in a single tube. Briefly, second-strand cDNA synthesis was conducted at 50°C (low stringency) during one cycle of first-stage PCR in a final reaction volume of 49.5 μl containing 3-5 μl (about 50 ng) of diluted first-strand cDNA, 5 μl of 10× PCR buffer plus Mg (Roche Applied Science, Mannheim, Germany), 5 μl of dNTP (each 2 mM), 1 μl of 10 μM dT-ACP2, and 1 μl of 10 μM arbitrary primer preheated to 94°C (Table 1). The tube containing the reaction mixture was held at 94°C, while 0.5 μl of Taq DNA Polymerase (5 U/μl; Roche Applied Science) was added to the reaction mixture. The PCR protocol for second-strand synthesis was one cycle at 94°C for 1 min, followed by 50°C for 3 min, and 72°C for 1 min. After the completion of second-strand DNA synthesis, 40 cycles were performed. Each cycle involved denaturation at 94°C for 40s, annealing at 65°C for 40 s, extension at 72°C for 40 s, and a final extension at 72°C to complete the reaction. The amplified PCR products were separated in 1.5-2% agarose gel stained with ethidium bromide.

### Cloning and sequencing

The differentially expressed bands were extracted from the gel using the QIAquick^® ^Gel extraction kit (QIAGEN Inc., Valencia, CA), and directly cloned into a TOPO TA^® ^cloning vector (Invitrogen) according to the manufacturer's instructions. The cloned plasmids were sequenced with an ABI PRISM^® ^3100 Genetic Analyzer (Applied Biosystems, Foster City, CA). Complete sequences were analyzed by searching for similarities using the Basic Local Alignment Search Tool (BLAST) search program at the National Center for Biotechnology Information GenBank [18].

### Quantitative reverse transcription - polymerase chain reaction (qRT-PCR)

All RT-PCR were set up in 96-well optical plates using 50 ng of extracted uninfected and infected RNA from CeLu, MDCK cell lines and chicken lung tissue, 10 μl TaqMan Universal PCR Master Mix (Applied Biosystems, Foster City, CA, USA), and 1 μl of primers/probe set containing 900 nM of forward and reverse primers and 300 nM probe was added to a final volume of 20 μl per reaction. All samples were tested in triplicates. RT-PCR program consisted of incubation at 48°C for 30 min, and 40 cycles at 95°C for 10 min, 95°C for 15 sec, and 60°C for 1 min with the Step One Plus Real-Time PCR System^® ^(Applied Biosystems). A non-template control and an endogenous control (eukaryotic 18s rRNA) were used for the relative quantification. All quantitations (threshold cycle [CT] values) were normalized to that of 18s rRNA to generate ΔCT, and the difference between the ΔCT value of the sample and that of the reference (uninfected sample) was calculated as ΔΔCT. The relative level of gene expression was expressed as 2^-ΔΔCT ^[19]. Primers for qRT-PCR were designed using Primer3 software (http://frodo.wi.mit.edu/cgi-bin/primer3/primer3.cgi) with these parameters: amplicon length, 95-100 bp; primer length, 18-27 nucleotides; primer melting temperature, 60-64°C; primer and amplicon GC content, 20-80%; difference in melting temperature between forward and reverse primers, 1-2°C. Primers were synthesized by Integrated DNA Technologies (Coralville, IA, USA). Primer information is listed in Table 2.

**Table 2:** Primers used in real time PCR assays

Amplification target	Sequence (5' - 3')
RTN4 F	TTACAGATTGCACTGCGTC
RTN4 R	TGTAACTGCACAAATCTACCCT
RTN4 probe	**6FAM**-GCTGCAGTTGTGCAGCAGGGACTGCA-**Iowa Black FQ**
L14 F	TCTTCCTTCTCGCCGAAC
L14 R	CATCAACCAAAGCCCTGTT
L14 probe	**6FAM**-TGGCCGGGTGGCCTACGTCTCCTTT-**Iowa Black FQ**
Hsp60 F	CATGGTGTGACCGTAGCAA
Hsp60 R	GGCAATAGAGCGTACCAAGA
Hsp60 probe	**6FAM**-ACGAAGAGGCTGGGGATGGCACCACT-**Iowa Black FQ**
CCND1 F	CAGAGGAGAACAAACAGATCAT
CCND1 R	AAATGAACTTCACATCTGTGGC
CCND1 probe	**6FAM**-AGCACGCGCAGACCTTCGTAGCGCT-**Iowa Black FQ**
CCND2 F	TAAGCTAGCCAGAGTTTTCTCA
CCND2 R	CATGAAATGAATTTGCACCTCG
CCND2 probe	**6FAM**-TGGGCCCCAAGGAGTCCCACGGAAT-**Iowa Black FQ**
CCND3 F	AGTCTCCTCCCTTCCCTTT
CCND3 R	AGTAGCGATCCAGGTAGTTC
CCND3 probe	**6FAM**-AGGAGCAGCGCTGCGAGGAGGAAGT-**Iowa Black FQ**
IL-8 F	CACTGTGAAAAATTCAGAAATCATTGTTA
IL-8 R	CTTCACCAAATACCTGCACAACCTTC
IL-8 probe	**6FAM**-AATGGAAACGAGGTCTGCTTAAACCCCAAG-**Iowa Black FQ**
18s rRNA F	AGCTCGTAGTTGGATTTCTGTTAATAATTTA
18s rRNA R	GCATATGCCTGCTTTAAGCACTCT
18s rRNA probe	**6FAM**-TTTCTCAAAGTAAAATTTCA-**Iowa Black FQ**

### Statistical analysis

Results expressed as 2^-ΔΔCT ^were reported as mean standard deviation and analyzed using paired student's t test. *P *values < 0.05 were considered statistically significant.

## Results

### Cellular transcripts from 3 different host systems regulated during infection with HPAI H5N1

**Figure 1:**
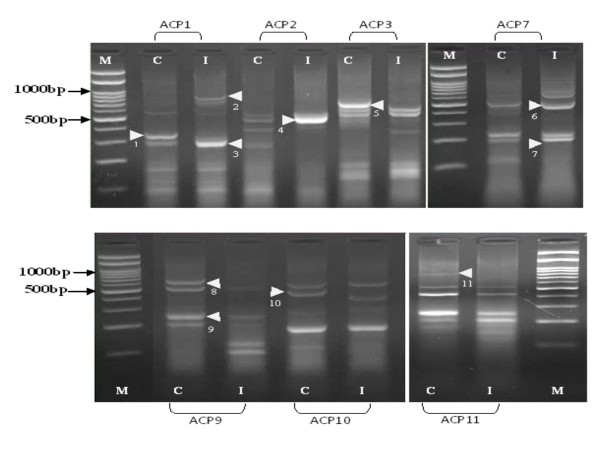
**There are 11 bands, of which 5 of them are up regulated and 6 down regulated.** The identities of these bands are presented in Table 3. M: 100 bp molecular marker; C: Control CeLu cell; I: CeLu cells infected with H5N1. Arrow indicates differential cDNA bands and each number indicates the DEG number.

**Figure 2:**
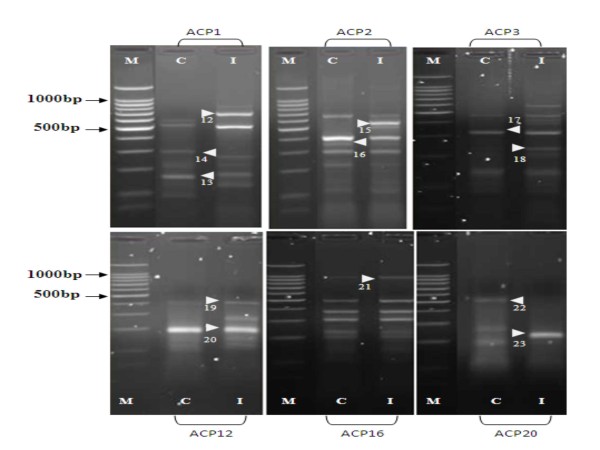
**There are 12 bands, of which 6 of them are up regulated and 6 down regulated**. The identities of these bands are presented in Table 4. M: 100 bp molecular marker; C: Control MDCK cell; I: MDCK cells infected with H5N1. Arrow indicates differential cDNA bands and each number indicates the DEG number.

**Figure 3:**
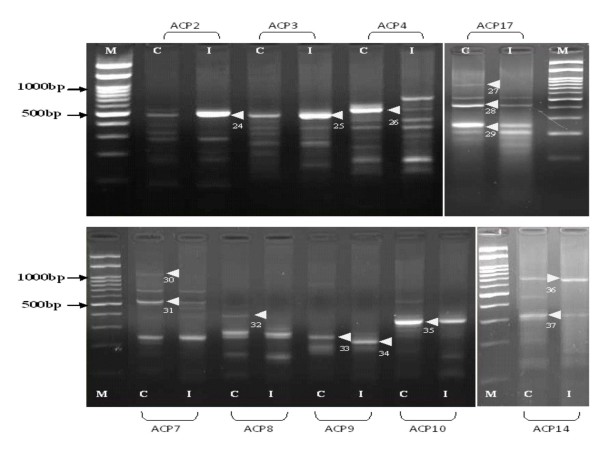
**There are 14 bands, of which 4 of them are up regulated and 10 down regulated**. The identities of these bands are presented in Table 5. M: 100 bp molecular marker; C: Control lung tissue; I: Lung tissue infected with H5N1. Arrow indicates differential cDNA bands and each number indicates the DEG number.

Differentially expressed mRNAs using a combination of 20 arbitrary primers and two anchored oligo (dT) primers (dT-ACP1 and dT-ACP2) of the 3 different types of host systems infected with the H5N1 virus were isolated, cloned and sequenced. For each of the host system, more than 100 transcripts were observed however, only distinct up regulated or down regulated transcripts as observed on the agarose gels were chosen i.e. after exclusion of poor bands and bands which did not show much differences in intensity between the control and infected cells. For the CeLu H5N1 infected cells (Figure 1) 11 distinct bands could be observed, of which 5 of the transcripts are up-regulated and 6 down-regulated. For the MDCK H5N1 infected cells (Figure 2), 12 distinct bands, of which 6 are up regulated and 6 down regulated were isolated. For the lung tissues of chickens infected with H5N1 virus (Figure 3), 14 bands that were significantly up-regulated (4 bands) or down-regulated (10 bands) from various annealing control primers were isolated. Using the BLAST database search, the 37 differentially expressed genes were identified i.e. 15 up-regulated and 22 down-regulated genes in the samples of infected hosts, compared to the uninfected. The functional roles, sequence similarities and characterization of the differentially expressed transcripts are summarized in Table 3, 4 and 5. BLASTn searches in GenBank revealed that the differentially expressed genes displayed significant similarities with known genes or expressed sequence tags (ESTs).

**Table 3:** Sequence similarities and characterization of differentially expressed transcripts of Chicken Embryo Lung Cells (CeLu)

Clone Name	Identity	Accession Number	Homology (%)	Putative functional role
DEG 1	PREDICTED: *Gallus gallus *similar to Prenyl (decaprenyl) diphosphate synthase, subunit 2 (LOC421777), mRNA (down regulated)	XM419804	96%	Catalyzes the formation of all trans-polyprenyl pyrophosphates from isopentyl diphosphate in the assembly of polyisoprenoid side chains, the first step in coenzyme Q biosynthesis. Defects in this gene are a cause of coenzyme Q10 deficiency.
DEG 2	*Dendrobium crumenatum *class-1 small heat shock protein mRNA, partial cds (up regulated)	EU302124	100%	Elevated temperatures or stress induced proteins, in which its expression is transcriptionally controlled.
DEG 3	*Clonorchis sinensis *clone CMXR-GP4 unknown mRNA (up regulated)	EU309047	100%	
DEG 4	Influenza A virus (A/open-billed Stork/Nakhonsawan/BBA0627May/05(H5N1)) polymerase PA (PA) gene, complete cds (up regulated)	EF112270	99%	Subunit of the trimeric complex of the RNA-dependent-RNA polymerase of Influenza A, involved in transcription of viral RNAs
DEG 5	*Clonorchis sinensis *clone ACP-1U-117-2 (down regulated)	EU780119	100%	
DEG 6	*Tigriopus japonicus *mitochondrial heat shock protein 60 (Hsp60) mRNA, complete cds; nuclear gene for mitochondrial product (up regulated)	EU306562	99%	Mitochondrial chaperonin that is typically held responsible for the transportation and refolding of proteins from the cytoplasm into the mitochondrial matrix. Involved in stress response from cells, especially during infection.
DEG 7	PREDICTED: *Gallus gallus *hypothetical LOC418666 (LOC418666), mRNA (up regulated)	XM416862	94%	
DEG 8	PREDICTED: *Gallus gallus *hypothetical LOC426258 (LOC426258), mRNA (down regulated)	XM423923	100%	
DEG 9	Unknown sequence (down regulated)			
DEG 10	Unknown sequence (down regulated)			
DEG 11	*Sus scrofa *calcium transporter 1 (TRPV6) mRNA, partial cds (down regulated)	FJ268731	100%	Membrane calcium channel which regulates calcium entry into the cell and also plays a role in calcium reabsorption.

**Table 4:** Sequence similarities and characterization of of Madine Darby Canine Kidney Cells (MDCK)

Clone Name	Identity	Accession Number	Homology (%)	Putative functional role
DEG 12	*Gibberella moniliformis *p450-4 gene for ent-kaurene oxidase, exons 1-4, strain A-00149 (up regulated)	AM946177	100%	Responsible for the synthesis of plant terpenoids.
DEG 13	*Pongo abelii *reticulon 4 (RTN4), mRNA >emb|CR861110.1| Pongo abelii mRNA; cDNA DKFZp459C0314 (from clone DKFZp459C0314) (down regulated)	NM001133403	90%	Protein that in humans is encoded by the RTN4 gene that has been identified as an inhibitor of neurite outgrowth specific to the central nervous system. It is also known to interact with Bcl-2 gene which is involved in promoting and inhibiting apoptosis in cells during stress.
DEG 14	*Homo sapiens *amyloid beta (A4) precursor protein, mRNA (cDNA clone IMAGE:4126584), (down regulated)	BC018937	94%	Neuronal adaptor protein that interacts with the Alzheimer's disease amyloid precursor protein (APP). It stabilizes APP and inhibits production of proteolytic APP fragments including the A beta peptide that is deposited in the brains of Alzheimer's disease patients
DEG 15	Influenza A virus (A/open-billed stork/Nakhonsawan/BBA0627 May/05(H5N1)) polymerase PA (PA) gene, complete cds (up regulated)	EF112270	99%	Subunit of the trimeric complex of the RN polymerase of Influenza A, involved in tra RNAs
DEG 16	unknown gene (down regulated)			
DEG 17	PREDICTED: *Canis familiaris *similar to ribosomal protein L14, transcript variant 2 (LOC480789), mRNA (down regulated)	XM537906	100%	Encodes a ribosomal protein that is a compon Involved in transcription
DEG 18	*Tigriopus japonicus *mitochondrial heat shock protein 60 (Hsp60) mRNA, complete cds; nuclear gene for mitochondrial product (up regulated)	EU306562	100%	Mitochondrial chaperonin that is typically transportation and refolding of proteins fr mitochondrial matrix. Involved in stre especially during infection.
DEG 19	TPA: TPA_inf: *Canis familiaris *RTN4-Cw (RTN4) gene, 3' UTR (up regulated)	BK003960	99%	Inhibitor of neurite outgrowth specific to t system.
DEG 20	PREDICTED: *Equus caballus *similar to cyclin D2 (LOC100051150), mRNA (down regulated)	XM001494152	94%	Belongs to the highly conserved cyclin family, whose members are characterized by a dramatic periodicity in protein abundance through the cell cycle. Cyclins function as regulators of cyclin-dependent kinases. Forms a complex with and functions as a regulatory subunit of CDK4 or CDK6, whose activity is required for cell cycle G1/S transition. Involved in the phosphorylation of tumor suppressor protein Rb
DEG 21	Mouse mRNA for amyloid beta precursor (protease nexin II) (up regulated)	X59379	95%	Up regulated during neuronal differentiati neural injury.
DEG 22	unknown gene (down regulated)			
DEG 23	*Bos taurus *reticulon 4 (RTN4), transcript variant 1, mRNA > gb|AY164744.2| *Bos taurus *RTN4-C (RTN4) mRNA, complete cds (up regulated)	NM001075138	88%	Inhibitor of neurite outgrowth specific to the central nervous system

**Table 5:** Sequence similarities and characterization of differentially expressed transcripts of Chicken Lung Tissues

Clone Name	Identity	Accession Number	Homology (%)	Putative functional role
DEG 24	Influenza A virus (A/open-billed stork/Nakhonsawan/BBA0627 May/05(H5N1)) polymerase PA (PA) gene, complete cds (up regulated)	EF112270	99%	Subunit of the trimeric complex of RNA polymerase of Influenza transcription of viral RNAs.
DEG 25	Messenger fragment for chicken pro-alpha-1 (I) collagen (up regulated)	V00401	96%	Encodes the major component of type I collagen, the fibrillar collagen found in most connective tissues, including cartilage, involved in cell proliferation.
DEG 26	*Gallus gallus *mRNA for hypothetical protein, clone 6l18 (down regulated)	AJ719809	97%	
DEG 27	Synthetic construct immunoglobulin heavy chain mu constant region and immunoglobulin heavy chain gamma 1 constant region genes, partial cds (down regulated)	EF495199	99%	Have variable domains which are important for binding with antigens.
DEG 28	*Clonorchis sinensis *clone CMXR-GP77 pyruvate dehydrogenase a-chain type 1 mRNA, partial **cds **(down regulated)	EU290665	100%	First component enzyme of pyruvate dehydrogenase complex, contributes to transforming pyruvate into acetyl-CoA by a process called pyruvate decarboxylation in citric acid cycle (cellular respiration).
DEG 29	unknown gene (down regulated)			
DEG 30	unknown gene (down regulated)			
DEG 31	unknown gene (down regulated)			
DEG 32	unknown gene (down regulated)			
DEG 33	*Sus scrofa *calcium Transporter 1 (TRPV6) mRNA, partial cds (down regulated)	FJ268731	100%	Membrane calcium channel which regulates calcium entry into the cell and also plays a role in calcium reabsorption.
DEG 34	PREDICTED: *Gallus gallus *hypothetical LOC418666 (LOC418666), mRNA (up regulated)	XM416862	94%	
DEG 35	*Rattus norvegicus *phosphodiesterase 7A (Pde7a), mRNA (down regulated)	NM031080	90%	
DEG 36	*Marmota monax *interleukin 8 (IL-8) gene, complete cds (up regulated)	EU332349	100%	Chemokine produced by macrophages and other cell types such as epithelial cells. It is one of the major mediators of the inflammatory response, particularly, serves as a chemical signal that attracts neutrophils at the site of inflammation.
DEG 37	*Crassostrea gigas *catalase mRNA, complete cds (down regulated)	EF687775	100%	

### Quantitative reverse transcription-PCR (qRT-PCR)

**Figure 4:**
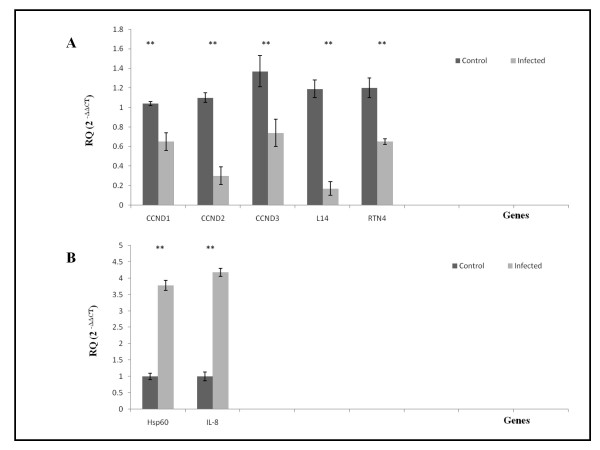
**Relative quantitation of down regulated and up regulated genes**. **A**. Relative quantitation of CCND1, CCND2, CCND3, L14 and RTN4 genes was determined by real time RT-PCR and the fold difference (2^-ΔΔCT ^) between the study groups were calculated: Uninfected cells (Control); Infected cells (Infected). **B**. Relative quantitation of Hsp60 and IL-8 genes was determined by real time RT-PCR and the fold difference (2^-ΔΔCT ^) between the study groups were calculated: Uninfected cells (Control); Infected cells (Infected). The **(*P *value) for both sets of data is <0.05, indicating there are significant down regulation and up regulation in the infected cells compared to control.

A real-time RT-PCR assay was developed to validate the mRNA differential display expression data based on the use of a TaqMan probe. We have chosen 7 genes (CCND1, CCND2, CCND3, RTN4, L14, Hsp60 and IL8) that were analyzed using qRT-PCR. Initially, we had intended to only measure the relative quantitation of the down regulated CCND2 (from the mRNA differential display results), however, we thought that it would be interesting to investigate whether H5N1 down regulates other cyclin D family (CCND1 and CCND3) as well. For optimal relative quantification of the 7 selected genes, the fold difference of ΔCT (2^-ΔΔCT ± SD^) between study groups were calculated (uninfected and infected cells). Resulting values were 1.04 ± 0.02 for CCND1 gene in uninfected cell, 0.65 ± 0.09 for CCND1 gene in infected cell, 1.10 ± 0.05 for CCND2 gene in uninfected cell, 0.30 ± 0.09 for CCND2 gene in infected cell, 1.37 ± 0.16 for CCND3 gene in uninfected cell, 0.74 ± 0.14 for CCND3 gene in infected cell, 1.19 ± 0.09 for L14 gene in uninfected cell, 0.17 ± 0.07 for L14 gene in infected cell, 1.20 ± 0.10 for RTN4 gene in uninfected cell, 0.65 ± 0.03 for RTN4 gene in infected cell (Figure 4A). These results indicate that there is a significant decrease (*P *< 0.05) in transcription of these 5 genes due to H5N1infection. On the contrary, the resulting values for Hsp60 gene in uninfected cell were 1.00 ± 0.10, 3.78 ± 0.15 for Hsp60 gene in infected cell, 1.00 ± 0.13 for IL-8 gene in uninfected cell and 4.18 ± 0.12 for IL-8 gene in infected cell (Figure 4B). These results indicate that there is significant increase (*P *< 0.05) in transcription of these 2 genes due to H5N1 infection.

## Discussion

Difficulty often arises in identifying a gene responsible for a specialized function during a certain biological stage because the gene is expressed at low levels, whereas the bulk of mRNA transcripts within a cell are highly abundant [20]. To screen DEG transcripts in low concentrations while minimizing the false positive results, it was reasonable to use a PCR-based technique. Moreover, it was possible to detect GeneFishing™ technology reaction products easily on ethidium bromide-stained agarose gel. This was supposed to greatly assist studies searching for genes that are expressed differentially in cells under various physiological stages or experimental conditions. In the past, several approaches have been used to compare levels of gene expression, e.g. RT-PCR and northern blot analysis. These approaches were limited to the analysis of one gene at a time, whereas other methods, such as subtractive hybridization or variations in the differential display techniques, can determine multiple expression patterns of predetermined sequences; the latter technique is very sensitive, but not quantitative [21]. Large numbers of expressed genes can also be investigated using nucleic acid microarrays. These arrays allow for scanning of large numbers of genes rapidly; however, these techniques have the relative disadvantage of being suitable only for analysis of a fixed number of predetermined gene sequences [22,23].

In this study, mRNA differential display technique was utilized to screen for regulated cellular transcripts during H5N1 infection in 3 different host systems. MDCK cells derived from a continuous (immortal) neoplastic cell line are one of the best mammalian cell models to study the infection of avian influenza virus as it has all the necessary receptors for virus attachment and also can be propagated in large amounts. It is interesting though to compare the responses to the same virus infections of two cell lines, one a continuous mammalian cell line and a primary chicken cell line. Cultured primary CeLu cells provide a more physiologically relevant environment for the molecular target under examination than that same target expressed in an "artificial" immortalized cell environment. This is notably the case with primary chicken embryo lung cells, for which the complex interplay of endogenously expressed ion channels, second messengers and other cell signaling proteins, can be better recapitulated than in transformed immortalized cell lines. It was hypothesized that intact lungs, which are the primary organ for the proliferation of the virus would probably generate some similar DEGs as the CeLu cells due to their similarity in the origin. However, the physiological environment and complex immune interactions of lungs from infected chickens reveal genes that are relevant in a physiological setting involving host responses components to infection. One of these genes is the IL-8. Hundreds of genes (Figures 1, 2 and 3) of the 3 host systems were expressed, out of which 37 DEGs up-and down-regulated were isolated and identified where 8 of them were unknown genes. For all the 3 host systems although the same 20 ACPs were used for amplification, not one of the proteins isolated in any one cell/host system is similar.

Overall, our studies have demonstrated that the responses of the three host systems to infections with the same virus were substantially different from each other. The functional roles, sequence similarities and characterization of differentially expressed transcripts are summarized in Table 3, 4 and 5. In this study, ACP-based RT-PCR results showed that most of the differentially expressed genes exhibited significantly higher sequence similarity (90 to100%) with known coding regions of genes. Munir et al., 2005 have showed that the regulation of host cell transcription following infection with a virus is an intricate phenomenon and may or may not be shared among viral agents; indicating that viruses that are evolutionarily closely related may not share similar mechanisms to regulate host gene expression and likely have their own signature patterns of altering host physiology during replication. In our study, we can also conclude that host cell responses or the regulation of host cell transcriptions of different permissible cell or host systems following infection with the same virus may have their own specific patterns for altering host gene expressions and may not share similar mechanisms and pathways.

One of the drawbacks in using this technique is the small number of DEGs generated that could be captured on agarose gels. In comparison to the large numbers of expressed genes that can be investigated using nucleic acid microarrays. Despite this, we found several interesting genes, which were predominantly and consistently up regulated only in infected cells both in cell lines and also lung tissues. They are the Hsp60 small heat shock proteins, Cyclin D2, Interleukin-8.

Hsp60 is a common cellular protein that assists in the correct folding of proteins and stabilizes unfolded labile proteins [24]. These functions maintain the activities of some cellular proteins and facilitate enzymatic maturation. The former is a well-known function of Hsp60 under stress conditions, and an example of the latter is activation of procaspase-3 and prion protein through conformational change by Hsp60 [25-27]. Functioning as a chaperonin in eukaryotes, Hsp60 assembles into a heptamer and has ATPase activity for the release of bound protein [25,28]. Hsp60 is an essential factor for the activation of human Hepatitis B Virus polymerase for it to function inside cellular environment [29]. Apart from that, Hsp60 as a mitochondrial protein has been shown to be involved in stress response as well. The heat shock response is a homeostatic mechanism that that protects cell from damage by up regulating the expression of genes that code for Hsp60 [30]. The up regulation of Hsp60 production allows for the maintenance of other cellular processes occurring in the cell, especially during stressful times. Infection and disease are extremely stressful on the cell. When a cell is under stress, it naturally increases the production of stress proteins, including heat shock proteins such as Hsp60. In order for Hsp60 to act as a signal it must be present in the extracellular environment. This explains the up regulated various types of heat shock protein and Interleukin-8 cytokine which we found during time of infection in our study. In addition to that, IL-8 is also a potent chemo-attractant and stimulus of neutrophils. It plays a pivotal role in inflammatory diseases. Hsp60 has also been found to be involved in signal transduction cascade of immune response when the cells are under stress from environment, in this case under viral attack. It acts as a signaling molecule, and indicates the action for other immune molecules such as cytokines (interleukins, interferons and also tumor necrosis factor) [31-34]. Further investigation on this Hsp60 also is essential, especially as it may shed some light on its role in cytokine storm that occurs in most H5N1 infection which is fatal to the host.

We also found the cyclin D2 transcript down regulated only during time of infection in MDCK cell lines which mimics a mammalian model of study. This cyclin forms a complex with and functions as a regulatory subunit of CDK4 or CDK6, whose activity is required for cell cycle G1/S transition. This protein has been shown to interact with and be involved in the phosphorylation of tumor suppressor protein Rb. G1 cell cycle regulators are often targets for deregulation in cancers [35,36]. Cyclin D2 is up regulated in many cancers, including breast cancer and its role is to increase cellular proliferation. In addition to cancer, the cyclin Ds are often seen deregulated in viral infections. For instance, cyclin D2 is up regulated in Epstein-Barr virus (EBV) and Hepatitis B virus (HBV) infected cells [37,38] and cyclin D1 is up regulated in Simian virus 40 (SV40) transformed cells [39]. It is interesting to note that EBV and HBV, both of which have increase levels, are associated with cancers. Many oncogenic viruses contain a viral protein, such as the SV40 T antigen and human papillomavirus (HPV) E6 and E7 proteins, which aid intumorigenesis by altering cell cycle progression [40,41]. Interestingly, in our study, we have found that, all 3 of the Cyclins, D1, D2 and D3 were down regulated. Very little is known about crosstalks between influenza A virus and the cellular machineries that regulate the cell cycle, thus our finding clearly opens up new avenues of research to determine whether alteration of cell cycle progression is a strategy used by H5N1 Avian Influenza virus to better replicate in host cells.

## Conclusion

The use of functional genomics methods, led by mRNA differential display technique, has significantly advanced our findings of organ and cell specific transcriptomes, especially when comparisons are made between infected and non-infected. We have managed to identify and isolate 37 authentic genes which were up and down regulated in this study. These findings in this preliminary study with MDCK and CeLu cell lines and lung tissues has open up new avenues of research especially into exploration and elucidating the functions of several interesting candidate genes such as the Hsp60, cyclin D2, IL-8 and the many unknown genes, and elucidating any role they might play in the virulence or pathogenicity of the virus. We have also shown that host systems infected by the same virus may have their own specific patterns for altering host gene expressions and may not share similar mechanisms and pathways. With further identification of these novel genes and the availability of sequence data of some of the unknown genes, would provide resources for further research in their use as markers or inhibitors in the development of novel biologics and reagents for diagnostic and anti-viral therapies.

## List of Abbreviations

MDCK: Madin Darby Canine Kidney; CeLu: Chicken Embryo Lung; HPAI: Highly Pathogenic Avian Influenza; AIV: Avian Influenza Virus; ACP: Annealing Control Primer; TCID: Tissue Culture Infective Dose; EID: Egg Infective Dose; DEGs: Differentially Expressed Genes; Hsp60: Heat Shock Protein 60, CDK4: Cyclin Dependant Kinase 4; CDK6: Cyclin Dependant Kinase 6; EBV: Epstein - Barr virus; HBV: Hepatitis B Virus; HPV: Human Papillomavirus; CCND1: Cyclin D1; CCND2: Cyclin D2; CCND3: Cyclin D3; RTN4: reticulon 4; L14: ribosomal protein L14; IL8: Interleukin 8.

## Competing interests

The authors declare that they have no competing interests.

## Authors' contributions

SSH conceived of the study. SSH, ARO, MM, SMN, RM and IO discussed the results. VRMTB and SSH performed the experiments, and VRMTB prepared the manuscript.

All authors have read and approved the final manuscript.

## Author's information

1. Assoc.Prof.Dr.Sharifah Syed Hassan (SSH)

Associate Professor - Microbiology

Doctor of Veterinary Medicine, UPM, Malaysia, 1983

MSc in Veterinary Microbiology, Univ. Surrey, UK, 1990

D. Phil Univ. of Oxford, UK, 1999

Dr.Sharifah joined Monash University, Sunway campus, Malaysia in March 2008 as an Associate Professor in Microbiology. She was the Director of Veterinary Research Institute (VRI), Malaysia from 2004 to 2007. She had extensive exposure and research experiences in areas of animal viral vaccine production, avian and mammalian virology diagnostics and research. Her main focuses of research are in production of recombinant proteins, viral and gene discovery for the development of novel diagnostic reagents and antiviral therapy. Her current work and research interests are in areas of cellular gene expression of virus infected cells especially Nipah and Avian Influenza Viruses.

2. Vinod RMT Balasubramaniam (VRMTB) finished his undergraduate degree in Asian Institute of Medicine, Science and Technology in 2007, majoring in Biotechnology. There, he produced several papers on plant genetic engineering, especially on *genetically engineered orchids which have resistant towards fungus*. He was accepted as research assistant under SSH in her laboratory. Subsequently, he was accepted to do PhD under SSH and currently undertaking the project, specially emphasizing on various host cellular genes infected with Avian Influenza Virus and their protein-protein interaction with viral genes.
